# Glutamate–Transporter Unbinding in Probabilistic Synaptic Environment Facilitates Activation of Distant NMDA Receptors

**DOI:** 10.3390/cells12121610

**Published:** 2023-06-12

**Authors:** Leonid P. Savtchenko, Dmitri A. Rusakov

**Affiliations:** UCL Queen Square Institute of Neurology, University College London, Queen Square, London WC1N 3BG, UK

**Keywords:** glutamate spillover, astrocyte, glutamate uptake, GLT-1, extrasynaptic actions, probabilistic synaptic model, Monte Carlo simulations

## Abstract

Once outside the synaptic cleft, the excitatory neurotransmitter glutamate is rapidly bound by its high-affinity transporters, which are expressed in abundance on the surface of perisynaptic astroglia. While this binding and the subsequent uptake of glutamate constrain excitatory transmission mainly within individual synapses, there is growing evidence for the physiologically important extrasynaptic actions of glutamate. However, the mechanistic explanation and the scope of such actions remain obscure. Furthermore, a significant proportion of glutamate molecules initially bound by transporters could be released back into the extracellular space before being translocated into astrocytes. To understand the implications of such effects, we simulated the release, diffusion, and transporter and receptor interactions of glutamate molecules in the synaptic environment. The latter was represented via trial-by-trial stochastic generation of astroglial and neuronal elements in the brain neuropil (overlapping spheroids of varied sizes), rather than using the ‘average’ morphology, thus reflecting the probabilistic nature of neuropil architectonics. Our simulations predict significant activation of high-affinity receptors, such as receptors of the NMDA type, at distances beyond half-micron from the glutamate release site, with glutamate–transporter unbinding playing an important role. These theoretical predictions are consistent with recent glutamate imaging data, thus lending support to the concept of significant volume-transmitted actions of glutamate in the brain.

## 1. Introduction

### 1.1. Glutamate Actions outside the Synaptic Cleft

Information handling and storage by the brain relies on rapid signal transfer and integration via excitatory circuits which use glutamate as their main neurotransmitter. Cell-to-cell glutamatergic synaptic connections have, thus, been considered an essential prerequisite for performing neural computations. A similar principle of one-to-one connectivity is at the core of theoretical neural network learning algorithms, which reflects the physical nature of wired electronic circuits. Consistent with this communication principle, remote actions of glutamate escaping the synaptic cleft of individual cell–cell connections are prevented via rapid binding to its high-affinity transporters, which are predominantly of the GLAST/GLT-1 type [1,2,3,4,5] and are expressed at high densities on astrocyte processes [6,7,8,9,10] that often permeate the perisynaptic neuropil [11,12,13,14,15]. However, experimental evidence has emerged suggesting that high-affinity glutamate receptors could be activated by synaptic discharges of glutamate, at least under repetitive or relatively strong stimuli, for up to a micron away from the release site [16,17,18,19,20,21,22,23,24]. This notion is consistent with recent observations that employed genetically encoded optical sensors to monitor glutamate released from individual axons in organised brain tissue [15,25,26]. Because excitatory synapses in the brain neuropil are only 0.5–0.8 μm apart, across species [27,28,29,30,31], extrasynaptic actions of glutamate could potentially constitute a significant volume-transmitted component of excitatory transmission. How this component is controlled by glutamate transporters and what the neurocomputational implications of such signalling are have remained an open question.

### 1.2. Glutamate Diffusion and Buffering in Probabilistic Synaptic Environment

Glutamate binding by its high-affinity transporters, such as GLT-1, occurs on the sub-millisecond scale, whereas the uptake itself, that is, the translocation step, takes tens of milliseconds [1,32]. Therefore, the stochastic nature of molecular reactions suggests that, in the case of GLT-1, approximately 35% of bound glutamate molecules could be released by their transporter molecules before being taken up [32]. Indeed, a ‘secondary’ wave of glutamate was predicted in detailed simulations of its release, diffusion, and uptake using detailed multicompartmental models [33]. A similar buffering phenomenon has been described in relation to genetically encoded optical glutamate sensors, which by definition release all initially bound glutamate molecules, thus slowing down their extracellular diffusion [34]. 

The complex phenomena of extrasynaptic glutamate diffusion, uptake, and buffering depend directly on the architecture of the synaptic environment and the expression patterns of glutamate transporters and other binding sites. However, attempts to model the fate of released glutamate have traditionally employed either contiguous-space approximation of the synaptic environment (with a space tortuosity factor) [18,19,28,34,35,36] or regular arrays of cubes or other shapes (e.g., [25,37,38,39]). In all such models, glutamate transporters are considered to be evenly distributed throughout the extracellular space. 

In reality, the synaptic environment is less deterministic. Firstly, the perisynaptic extracellular space is highly heterogeneous, as represented by a system of gaps, channels, and dead ends [38,40] of variable shapes and sizes [40,41,42]. Secondly, glutamate transporters are expressed primarily by perisynaptic astroglial processes, which occupy only 8–10% of neuropil volume in the hippocampus (~30% in the cerebellum) [15,33,43,44]. Finally, the morphology of perisynaptic extracellular space and the occurrence of transporter-enriched astroglia both vary strongly from synapse to synapse. The latter makes the concept of an ‘average synaptic environment’ somewhat unsatisfactory because the process of shape averaging effectively removes, or ‘chisels out’, any heterogeneities or outstanding features of individual environments. The latter features, however, could potentially determine the key characteristics of extrasynaptic glutamate activity. 

Thus, we attempted to introduce a simulation platform that models porous synaptic neuropil as a random scatter of overlapping spheres with a distributed size (that reflects 3D electron microscopic observations) [45,46,47]. The resulting ‘voids’, which vary arbitrarily in shape and size, represent the tortuous extracellular space occupying the corresponding tissue volume fraction (ɑ). Importantly, rather than having one fixed ‘average’ extracellular environment, each simulation run that tracks the release and diffusion of glutamate particles generates this environment anew. Thus, the effects of ‘outliers’, such as transporter-free diffusion escape routes, could be preserved in the average outcome of multiple space realisation runs. 

Our earlier attempt to simulate glutamate release and diffusion with this model generated virtually omnipresent transporter-enriched perisynaptic astroglia, with little chances of having diffusion escape routes [46]. While this arrangement might reflect the case of glutamatergic cerebellar synapses encircled by Bergmann glia [48], or synapses inside synaptic glomeruli [49], it is less representative of the common excitatory synapses occurring in cortical neuropil. The simulation paradigm was, therefore, developed further by assigning, in an arbitrary fashion, the roles of neuronal and astroglial elements to individual simulated spheroid shapes in accordance with their corresponding tissue volume fractions, as established empirically [47]. In that model, the interaction between diffusing glutamate molecules and transporter-enriched ‘astroglial’ spheroids was simulated as a stochastic binding event that may occur, with the probability operator incorporating both the experimental transporter affinity and their surface density, as further explained below. Introducing these realistic parameters revealed clear theoretical plausibility for a released glutamate to diffuse over a significant distance from its synaptic release site [47]. However, high demand for computational resources limited our previous exploration of glutamate escape of up to ~3 ms post-release [47], which is the time period before any significant glutamate–transporter unbinding in the extracellular space could occur [32,33]. In the present study, we attempted to understand how incorporating such multi-faceted factors in the model would affect the predicted activation of high-affinity glutamate receptors, such as of the NMDA type, outside active synapses.

## 2. Materials and Methods

### 2.1. Generating Probabilistic Synaptic Environment

**Figure 1:**
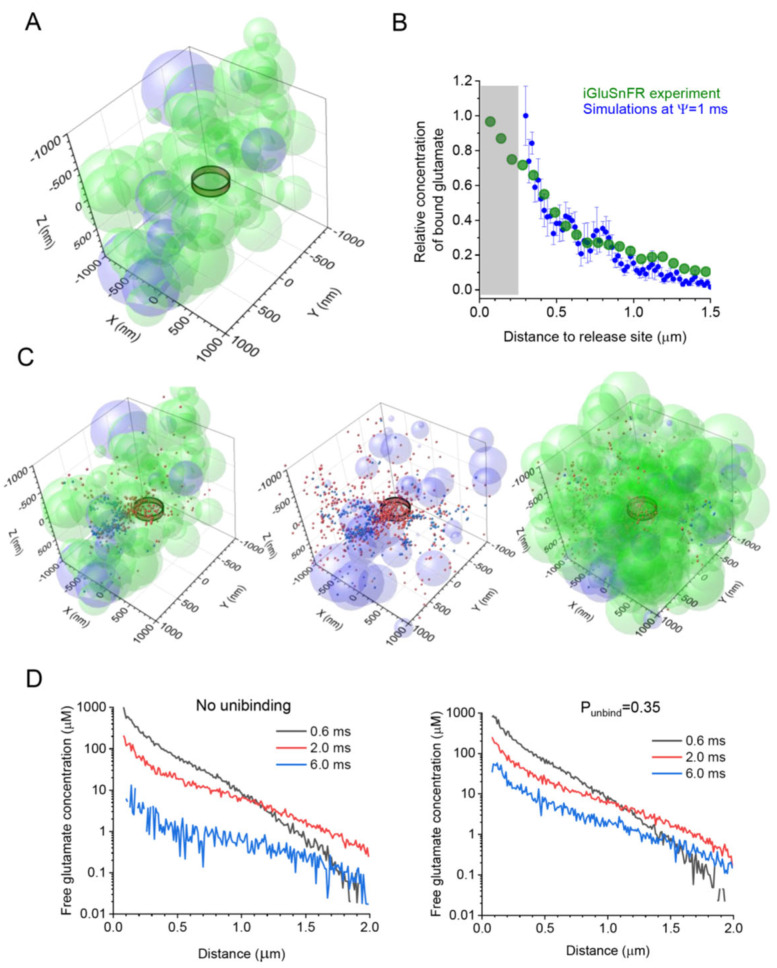
Simulating extrasynaptic glutamate escape and transporter binding and unbinding using a stochastic model of probabilistic synaptic environment. (**A**) Diagram depicting the extracellular space filled with overlapping spheroids representing neuronal (light green) and astroglial (light magenta) structures, with an extracellular space volume fraction ɑ = 0.2, and an astroglial volume fraction VF_astro_ = 0.1; the ring illustrates approximately the extent of the synaptic cleft (with two hemispheric obstacles representing pre- and postsynaptic elements [33]); and a tissue fragment (2 × 2 × 1 μm^3^ slab of the 4 × 4 × 4 μm^3^ simulation arena) is shown for presentation clarity. (**B**) The average spatial profile of iGluSnFR fluorescence (green) with respect to the glutamate release site (zero distance) near an individual CA3-CA1 synapse at its peak post-release, as recorded earlier [26], and the average profile (mean ± SEM, *n* = 10) of the simulated transporter-bound glutamate concentration (blue) at 4 ms post-release, for Ψ = 1 ms, as indicated. (**C**) Diagram as in A, but shown, for clarity, with astroglial transporter-bound (red) and free (blue) glutamate molecules (1000 particles, 1 ms post-release) using three presentations: as a space slab similar to A (left); with astroglial elements only (centre); and as a 2 × 2 × 2 μm^3^ arena filled with both neuronal and astroglial elements (right). (**D**) Simulated spatial profiles of free glutamate concentration at various time points post-release, as indicated, with no transporter unbinding (left) and with unbinding at a probability *P*_unbind_ = 0.35 (right).

Similar to the previous study [47], Monte Carlo simulations were carried out over a cube arena of 4 μm wide. In the arena centre, 1000 or 2000 Brownian particles representing glutamate molecules were released instantaneously into the synaptic cleft (release constrained within a 120 nm wide, 20 nm high cylindrical volume, and the rest of the cleft being made up by the adjacent extracellular space). The space outside the cleft was filled with randomly scattered, overlapping sphere shapes, with the smallest gap between the cleft and the nearest sphere constrained at ~10 nm. This was proceeded by generating random 3D coordinates of sphere centroids within the simulation arena and a random radius value for each sphere: the latter was distributed uniformly between 50 and 300 nm to roughly reflect the sizes of cellular elements seen in 3D EM reconstructions of the synaptic neuropil [38,50]. This space generation procedure was carried out anew for every new simulation run. The extracellular tissue volume fraction, ɑ, was validated in each simulation run by (i) distributing randomly the ‘test points’ over the 3D arena and (ii) calculating their fraction falling inside the spheres [46,47]. In the present study, the value of ɑ was explored between 0.1 and 0.3 [51,52,53]. Similarly, each generated spheroid was assigned the role of either a neuronal or an astroglial element, with the probability reflecting their respective tissue volume fraction (VF) that was established empirically (Figure 1A), such as VFs of ~0.1 and ~0.3 occupied by astrocyte processes in the hippocampal and cerebellar neuropil, respectively [33]. The diffusion coefficient of glutamate in the free extracellular space was set at *D* = 0.5 μm^2^/ms, in accordance with the in situ measurements of extracellular diffusivity in brain slices using time-resolved fluorescence anisotropy imaging [54]. 

### 2.2. Glutamate Binding to and Unbinding from Astroglial Surfaces

The interaction of glutamate and ‘neuronal’ spheroids was simulated as an elastic (mirror reflection) collision. In the case of ‘astroglial’ spheroids enriched in high-affinity transporters, the interaction was simulated for individual diffusing particles as a stochastic binding event that occurs with probability *P*. For each particle, *P* is a function of time *t* elapsed from the particle’s first collision with an ‘astroglial’ spheroid in accordance with the classical lifetime expression for first-order reactions, *P* = 1 − exp(−*t*Ψ^−1^). Here, Ψ is the time constant (free parameter) that determines how soon, on average, the forthcoming binding event occurs when a particle remains near an astroglial surface. Parameter Ψ, thus, combines, in a single quantity, the effects of the transporter binding affinity, the transporter cell surface density, and the proximity of the binding surface to the diffusing particle. During the simulations, *P* was computed at every diffusion time step [32] as long as a diffusing particle remained within 5 nm of an ‘astroglial’ spheroid. *P* was reset to zero once the particle departed from the ‘astroglial’ surface by >5 nm. We tested that increasing the cut-off distance above 5 nm in our conditions had no detectable effect on *P*. 

To constrain the value of Ψ, we first analysed the data from our previous experiments reporting the spatial profile of glutamate bound to the optical glutamate sensor iGluSnFR, following its release from a single axonal bouton, near the fluorescence signal peak [26], as explained in the results. The expected time constant of glutamate unbinding from GLT-1 is ~4 ms, with the expected glutamate unbinding probability (as opposed to glutamate translocation into an astrocyte) of ~0.35 [32]. To introduce the effect of unbinding into our simulations, glutamate molecules that were bound to transporters were released into the extracellular space with the time-dependent probability that followed an S-function (cumulative Gaussian distribution, σ = 2 ms) crossing 0.5 at *t* = 4 ms, in accordance with the approximate unbinding lifetime statistics. Once unbound, glutamate molecules were allowed to diffuse freely and bind to their transporters repeatedly if encountered. Because the time of simulation (<10 ms) was much shorter than the typical time for glutamate to be transported inside an astrocyte [32], the latter was not considered.

### 2.3. Kinetics of Free Glutamate and NMDA Receptor Activation

Typically, each test run lasted for 9–10 ms (system time) post-release, with 1000 diffusing Brownian particles (glutamate molecules) tracked at every time step. Following 10 or 20 (as specified) runs, the average numbers of bound and free glutamate molecules were calculated within the 20 nm thick concentric shells centred at the release site, at required time points. The corresponding particle numbers and extracellular space volumes provided the absolute concentration values. It has been estimated that approximately 12,000 molecules of the main astroglial glutamate transporter, GLT1, normally occur per 1 μm^3^ of hippocampal neuropil [10]. In such non-saturating conditions (unlimited supply of transporters even in the vicinity of the synapse), the concentration time course of a much smaller glutamate amount will scale linearly with the number of molecules. Thus, assuming that the average synaptic vesicle contains ~3000 glutamate molecules, the resulting concentrations were multiplied by a factor of 3. 

The glutamate concentration time course obtained this way was used as the initial conditions to generate NMDA receptor kinetics (5 channel states) [55] by solving the corresponding system of differential equations, as shown previously [28,39,55], for different distances from the release site, using the MATLAB built-in ‘ode45’ function with an accuracy of 10^−8^. Monte Carlo simulations were run using two computing environments. The initial testing used a UCL Myriad cluster: processors per node, Intel(R) Xeon(R) Gold 6240 CPU @ 2.60 GHz; cores per node of 36 + 4 A100 GPUs; and RAM per node of 192 GB, tmpfs 1500 G, with a total of 6 nodes. The second environment was cloud computing with Amazon AWS: t4g.medium, with a memory of 4 GB. Parallelisation and optimisation of the algorithms and program codes were implemented by AMC Bridge LLC (Waltham, MA, USA), Unboltsoft (Dnipro, Ukraine), with internet security assistance provided by Cybecurio Ltd. (Berkhamsted, UK). 

The kinetics of the glutamate transporter GLT-1 involving glutamate and ion fluxes have been incorporated into our ASTRO simulation platform [56] and are available online (description in the GluTrans.mod file) at https://github.com/LeonidSavtchenko/Astro/tree/master/neuronSims (accessed on 7 April 2023). The NMDAR kinetics scheme that we and many others use is available from the mod file in the NEURON-ModelDB platform at https://senselab.med.yale.edu/modeldb/ShowModel?model=18198&file=/SYN_NEW/nmda5.mod#tabs-2 (accessed on 7 April 2023), with the set of parameters in accordance with [55]. 

## 3. Results

### 3.1. Constraining the Glutamate–Transporter Binding Parameter Ψ

GLT-1 transporters and the optical glutamate sensor iGluSnFR display their binding rates within a similar diffusion-limited range, and neither is saturated by individual glutamate release events [3,26,57,58]. Therefore, their interactions with glutamate should be similar on the small millisecond scale [34]. Based on this notion, we sought to compare the experimental and simulated data reporting the spatial concentration profiles of glutamate bound to either iGluSnFR (experimental) or GLT-1 (simulated) following its release into the synaptic cleft. Thus, as a first approximation, we compared the profile of iGluSnFR fluorescence (near its peak) recorded following a single-synapse glutamate discharge in an earlier study [26], with the concentration profile of bound glutamate simulated using the present model (a snapshot of 4 ms post-release, near the peak of binding), while varying the value of Ψ between 0.1 and 10 ms. The best fit between the simulated and the experimental profiles of glutamate (bound to either GLT-1 or iGluSnFR molecules) corresponded to Ψ~1 ms (Figure 1B). This value was, therefore, used in subsequent simulations.

### 3.2. Simulating the Dynamics of Glutamate in Probabilistic Environment

We started by simulating a synaptic environment that would possess the main morphometric parameters of hippocampal neuropil (mainly area CA1) obtained experimentally. This included an extracellular space volume fraction ɑ of ~0.2 [41,59], an astroglial volume fraction VF_astro_ of ~0.1 [15,33], and a ɑ ~250 μm wide synaptic apposition area including the cleft (a ~1 μm slab of simulated tissue is depicted in Figure 1A). An instantaneous release of Brownian particles (glutamate molecules) into the clef centre generated a time-dependent diffusion and astroglia-binding scatter, with the 3D co-ordinates of all individual molecules, either freely diffusing or bound, traced and recorded throughout the system time (Figure 1C; same scatter shown with partially complete representations of the tissue environment for illustration purposes). 

In each simulation run, we calculated the concentration of both free or bound glutamate within 20 nm thick concentric shells centered at the release site, at varied distances from the site, and at different time points post-release. We noted that every individual stochastic generation of the synaptic environment produced a relatively unique geometric configuration (albeit with the same VF values for the extracellular space and astroglia), adding to the variability of the glutamate concentration profile. Thus, to obtain the ‘average profile’, we carried out 10 such realisations, generating both the tissue environment and glutamate diffusion in it, and calculated the average concentration readout. The results were used to monitor and compare the free-glutamate profile under no unbinding from transporters (Figure 1D, left) and under an unbinding probability of 0.35 (Methods; Figure 1D right).

### 3.3. Glutamate–Transporter Unbinding Facilitates NMDAR Activation at >500 nm from Release Site

**Figure 2:**
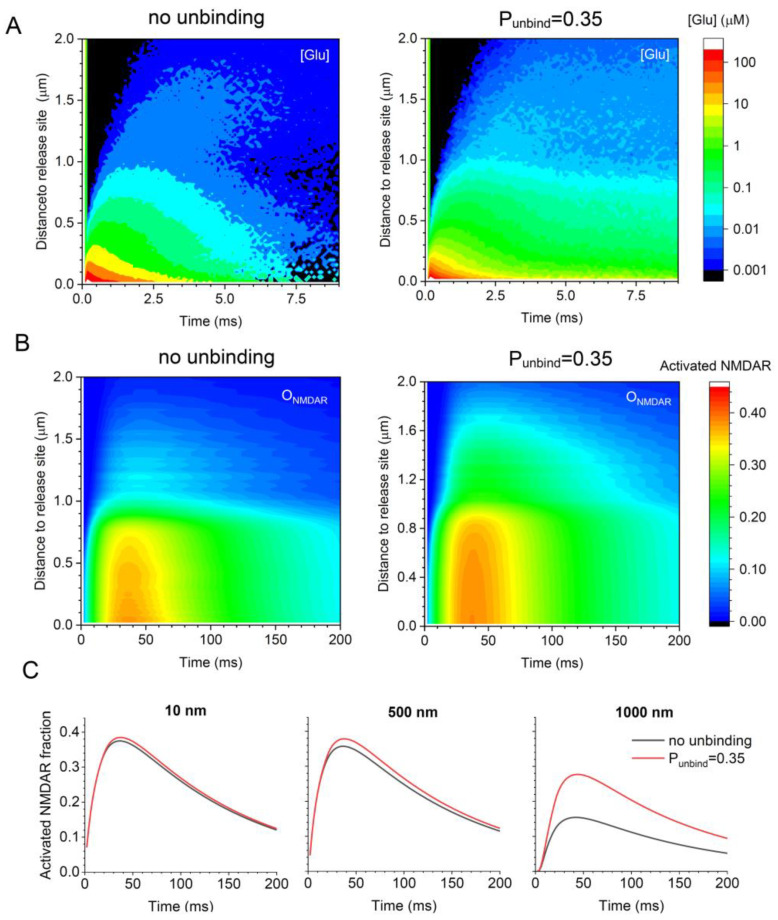
Glutamate–transporter unbinding facilitates NMDAR activation away from the release site at the micron scale. (**A**) Diagrams illustrating the free-glutamate concentration kinetics (false colour scale) at different distances from the release site, with no transporter unbinding and with unbinding with P_unbind_ = 0.35, as indicated. (**B**) Diagrams illustrating NMDAR activation kinetics (assuming no Mg^2+^ block) under the conditions shown in (**A**). (**C**) Examples of the NMDAR activation time course at three different distances from the release site, with (red) and without (grey) transporter unbinding, as indicated and shown for comparison. Key model parameters: ɑ = 0.2, VF_astro_ = 0.1, and Ψ = 1 ms.

We obtained the time–distance maps for the free-glutamate concentration dynamics in the ‘baseline’ case (ɑ = 0.2 and VF_astro_ = 0.1), with and without glutamate–transporter unbinding being enabled (Figure 2A). These data generated the corresponding maps for the NMDAR activation (Figure 2B), under relieved Mg^2+^ block (see Discussion). Firstly, the results predict significant activation (or at least double occupancy) of NMDARs in the probabilistic synaptic environment at up to 0.5 μm from the release site (Figure 2C, left and centre). Secondly, at distances up to 1 μm form the synapse centroid, glutamate–transporter unbinding appears to increase NMDAR activation by almost two-fold (Figure 2C).

### 3.4. Glutamate–Transporter Unbinding Makes a Difference under Varied Synaptic Environments

**Figure 3:**
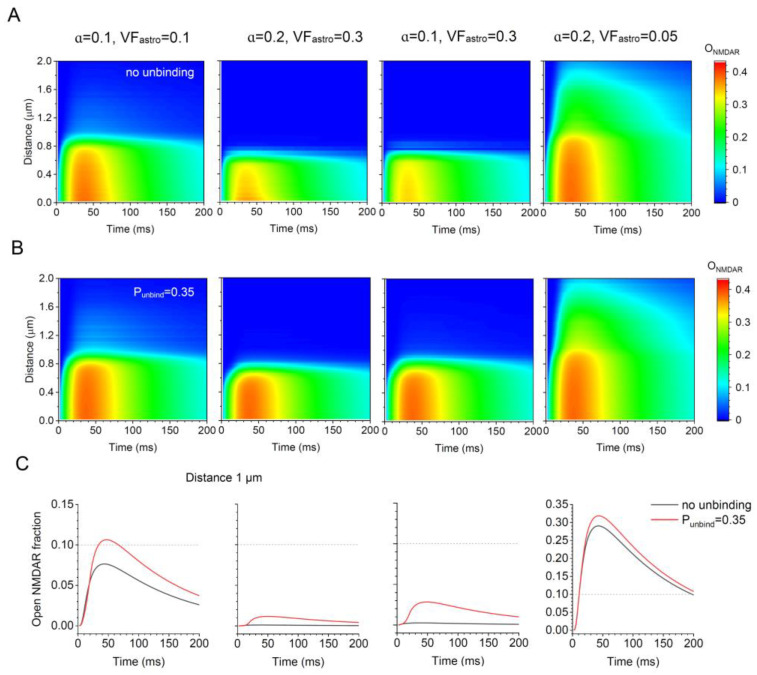
Glutamate–transporter unbinding facilitates NMDAR activation under varied parameters of the synaptic microenvironment. (**A**) Diagrams illustrating simulated NMDAR activation kinetics (assuming no Mg^2+^ block) under the values of ɑ and VF_astro_, as indicated, with no glutamate-transporter unbinding. (**B**) Diagrams as in A, with glutamate–transporter unbinding occurring with P_unbind_ = 0.35. (**C**) Examples of the NMDAR activation time course at 1 μm from the release site, with (red) and without (grey) transporter unbinding, as indicated and shown for comparison. The values of ɑ and VF_astro_ are shown at the top.

Finally, we examined if the effect of glutamate–transporter unbinding on NMDAR activation remained detectable when both astroglial presence and the key features of the synaptic environment varied within the known range. We, therefore, carried out our simulations under the condition of reduced extracellular space (ɑ = 0.1) or reduced expression (occurrence on the astrocyte surface) of glutamate transporters (V_astro_ = 0.05), which is believed to take place in some pathological conditions [60,61,62], and under the condition of an increased astroglial VF (VF_astro_ = 0.3), such as in the cerebellum [33]. The results indicate, firstly, that increasing the presence of transporter-enriched astroglia sharply reduces the chance of NMDAR activation beyond 0.5 μm from the release site, whereas changes in the extracellular space fraction appear to have a lesser effect (Figure 3A). Secondly, in all the cases considered here, glutamate unbinding increases the activation of NMDARs at various distances beyond 0.4.-0.5 μm (Figure 3A,B). Interestingly, the latter effect is particularly prominent at distances as large as 1 μm from the release site (Figure 3C). 

## 4. Discussion

The traditional neuroscience view has been that informative signal propagation and memory formation in the brain occurs mainly through the activity of neuronal networks equipped with point-to-point, or ‘wired’, excitatory connections. However, the main excitatory neurotransmitter glutamate, once released from a synaptic vesicle, rapidly leaves the synaptic cleft; several thousands of released molecules bind to no more than a hundred or two hundreds of target receptors or transporters within the cleft. The escaping glutamate molecules could, therefore, activate their extrasynaptic receptors, at least in theory, and thus compromise connection specificity. To counter such effects, many excitatory synapses are surrounded by astroglial processes that express high-affinity glutamate transporters, such as GLT-1. However, electron microscopy studies indicated that such processes cover only a proportion of synapses and that astroglial coverage of individual synapses is often incomplete [12,13,14,63]. Thus, there often remain perisynaptic diffusion escape routes for glutamate, which could potentially lead to the activation of its target receptors away from the synapse. However, theoretical models that test this possibility normally consider an ‘average’, hence homogeneous, spatial distribution of extracellular diffusivity and high-affinity transporters in the synaptic environment. The latter, even though diluting the transporter density to fill the entire perisynaptic space, would still appear sufficient to block, at least theoretically, longer-range escape of glutamate. 

The present study attempts to abandon the idea of the ‘average’ environment, preferring instead an exploration of the probabilistic extracellular space that varies from synapse to synapse [47]. Thus, every modelled realisation of the perisynaptic environment may leave significant diffusion escape routes devoid of astroglial membranes and, hence, glutamate transporters. When implemented, such an approach predicts significant presence of escaping glutamate up to 1 μm from its release site. In fact, this prediction appears consistent with recent experimental data documenting a significant signal generated by the glutamate sensor iGluSnFR at similar distances from an individual activated synapse [25,26]. 

Thus, under the conditions of a probabilistic synaptic environment, we attempted to evaluate the chances for glutamate molecules to activate NMDARs at various distances from their release site. We found that, firstly, such activation is plausible, assuming no Mg^2+^ block, beyond an average nearest-neighbour inter-synaptic distance (~0.5 μm in the hippocampal neuropil [28]). In fact, the data represent the kinetics of NMDAR double occupancy by glutamate, leading to NMDAR activation if a host cell becomes depolarised above approximately -50 mV. Thus, glutamate discharge by one synapse can result in the NMDARs at some neighbouring cells being doubly occupied, or ‘tagged’, by glutamate for up to 150–250 ms. The ‘tagged’ NMDARs can be activated by host cell depolarisation or spiking, without any synaptic input at the location of ‘tagged’ NMDARs. Because of the relatively close inter-synaptic distances in the neuropil, these data predict that a significant proportion of synapses could carry ‘tagged’ NMDARs during network activity. This may have important repercussions for signal integration rules in the brain. Secondly, because of stochastic unbinding of glutamate molecules from transporters [32,34], our simulations predict significant NMDAR activation up to 1 μm from the synapse. With a synaptic density of ~2 μm^−3^, this distance would reach approximately eight synaptic neighbours, thus raising questions about the contribution of volume-transmitted glutamate signal to the excitatory activity of brain networks. Notably, a reduction in the expression of the main astroglial glutamate transporter GLT1, which is characteristic for some common neurological conditions [61,64], could expand dramatically the volume-transmitted actions of glutamate (Figure 3). 

The theoretical approach outlined here is not devoid of limitations. Firstly, while overlapped spheroids appear more realistic than regular geometric shapes when modelling the tortuous and variable extracellular space, they are still not equivalent to natural cellular forms. Secondly, the exact value of glutamate–transporter unbinding probability P_unbind_ requires further experimental validation, although an exploration of the plausible range of kinetic constants has indicated that this value probably has a lower estimate [32]. Finally, we assumed no Mg^2+^ block of NMDARs to evaluate their activation kinetics, which is unlikely to occur continuously in the brain neuropil. However, NMDARs that are double bounded by glutamate for hundreds of milliseconds after glutamate release would be ‘primed’ for activation, if and when the host cell is depolarised. Thus, long-range actions of glutamate might provide a time window to lower the threshold of spatial integration for excitatory signals and their NMDAR-dependent plasticity [39]. Finally, it would seem critical to establish whether the volume-transmitted extrasynaptic glutamate actions occur to a similar extent in the intact brain. 

## 5. Conclusions

Recent experimental studies have reported that optical sensors can detect escaping glutamate more than half-micron away from an individual synapse that discharge it. This is difficult to explain mechanistically using the traditional detailed models of an ‘average’ synaptic environment that incorporates experimentally established prevalence of astroglial glutamate transporters. Here, we modelled the synaptic environment using trial-by-trial stochastic generation of astroglial and neuronal elements to reflect highly probabilistic configurations of the perisynaptic extracellular space. It appears that the presence of occasional transporter-free escape routes around synapses, and the relatively rapid transporter-unbinding kinetics of glutamate, may lead to the significant occupancy of high-affinity glutamate receptors at micron-scale distances from active synapses. Establishing an adaptive role for such volume-transmitted glutamate signalling in the brain is an important and intriguing question. 

## Data Availability

All original data are available upon request.
