# Correction: Savtchenko, L.P.; Rusakov, D.A. Glutamate–Transporter Unbinding in Probabilistic Synaptic Environment Facilitates Activation of Distant NMDA Receptors. *Cells* 2023, *12*, 1610

**DOI:** 10.3390/cells14131003

**Published:** 2025-07-01

**Authors:** Leonid P. Savtchenko, Dmitri A. Rusakov

**Affiliations:** UCL Queen Square Institute of Neurology, University College London, Queen Square, London WC1N 3BG, UK

In the original publication [1], there was an error in Figure 1D that was caused by a misprint—the corrected values were not automatically saved in the PDF file. The time labels should be corrected as follows: instead of 0.3 ms, 1.0 ms, and 3.0 ms, they should read 0.6 ms, 2.0 ms, and 6.0 ms, respectively, in both graphs. The correct Figure appears below:

**Figure 1:**
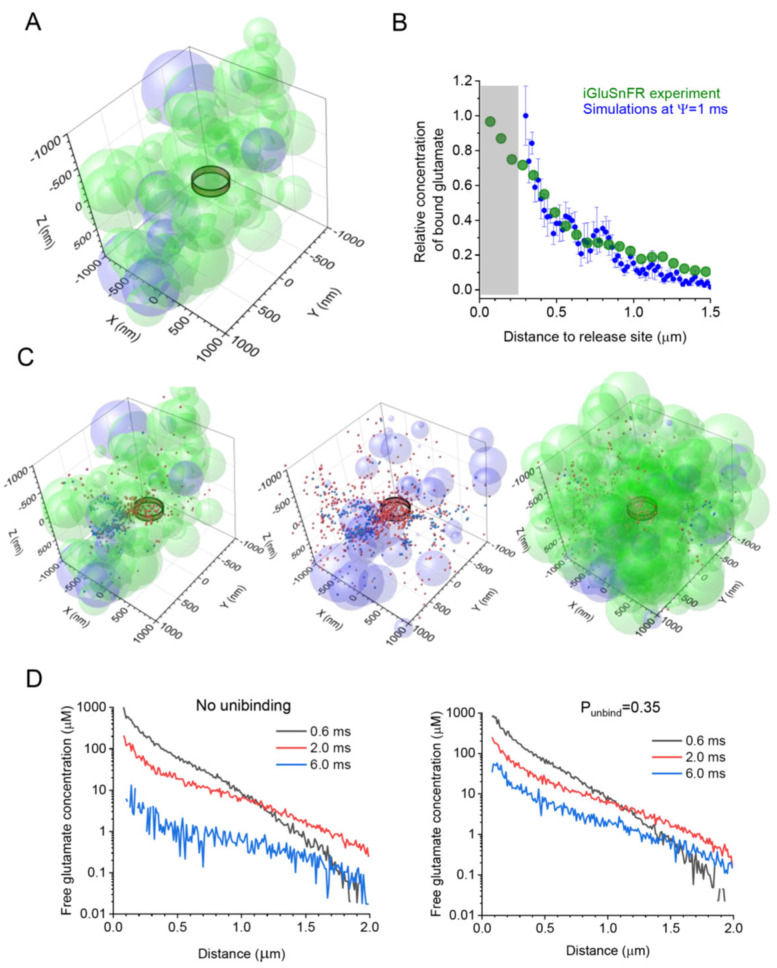
Simulating extrasynaptic glutamate escape and transporter binding and unbinding using a stochastic model of probabilistic synaptic environment. (**A**) Diagram depicting the extracellular space filled with overlapping spheroids representing neuronal (light green) and astroglial (light magenta) structures, with an extracellular space volume fraction α = 0.2, and an astroglial volume fraction VF_astro_ = 0.1; the ring illustrates approximately the extent of the synaptic cleft (with two hemispheric obstacles representing pre- and postsynaptic elements [33]); and a tissue fragment (2 × 2 × 1 μm^3^ slab of the 4 × 4 × 4 μm^3^ simulation arena) is shown for presentation clarity. (**B**) The average spatial profile of iGluSnFR fluorescence (green) with respect to the glutamate release site (zero distance) near an individual CA3-CA1 synapse at its peak post-release, as recorded earlier [26], and the average profile (mean ± SEM, *n* = 10) of the simulated transporter-bound glutamate concentration (blue) at 4 ms post-release, for Ψ = 1 ms, as indicated. (**C**) Diagram as in A, but shown, for clarity, with astroglial transporter-bound (red) and free (blue) glutamate molecules (1000 particles, 1 ms post-release) using three presentations: as a space slab similar to A (left); with astroglial elements only (centre); and as a 2 × 2 × 2 μm^3^ arena filled with both neuronal and astroglial elements (right). (**D**) Simulated spatial profiles of free glutamate concentration at various time points post-release, as indicated, with no transporter unbinding (left) and with unbinding at a probability *P*_unbind_ = 0.35 (right).

The authors state that the scientific conclusions are unaffected. This correction was approved by the Academic Editor. The original publication has also been updated.
